# Risk Factors for Postoperative Pancreatic Fistula After Pancreaticoduodenectomy: A Single-Center Experience

**DOI:** 10.7759/cureus.110526

**Published:** 2026-06-09

**Authors:** Ahmed Reza Suny, Sadia Islam, Lokman Hossain, Muhtasim Mustafa Sium, Ashraf Siddik Adel

**Affiliations:** 1 Department of Hepatobiliary Surgery, Dhaka Medical College Hospital, Dhaka, BGD; 2 Department of Surgery, Mugda Medical College and Hospital, Dhaka, BGD; 3 Department of Surgery, Shaheed Suhrawardy Medical College and Hospital, Dhaka, BGD; 4 Department of Surgery, Dhaka Medical College Hospital, Dhaka, BGD; 5 Department of Surgery, BIRDEM General Hospital, Dhaka, BGD

**Keywords:** pancreatic duct diameter, pancreaticoduodenectomy, pancreatico-jejunostomy, postoperative pancreatic fistula, risk factors, serum albumin

## Abstract

Background: Postoperative pancreatic fistula (POPF) is a common and clinically significant complication following pancreaticoduodenectomy (PD), contributing to increased morbidity, prolonged hospitalization, and mortality. This study aimed to determine the incidence and independent predictors of POPF in a tertiary care setting in Bangladesh.

Methods: This prospective observational study included 100 consecutive patients who underwent PD at Dhaka Medical College Hospital from January to December 2024. Patients were classified into POPF and non-POPF groups based on the 2016 International Study Group of Pancreatic Surgery (ISGPS) criteria. Preoperative, intraoperative, and postoperative variables were analyzed. Multivariate logistic regression was performed to identify independent predictors of POPF.

Results: POPF occurred in 28% of patients, with clinically relevant POPF (Grade B/C) accounting for 18%. On multivariate analysis, soft pancreatic texture (adjusted OR: 5.21, 95% CI: 1.73-15.68), pancreatic duct diameter < 3 mm (adjusted OR: 4.36, 95% CI: 1.52-12.47), serum albumin < 32 g/L (adjusted OR: 3.02, 95% CI: 1.11-8.19), elevated CRP > 20 mg/L (adjusted OR: 2.94, 95% CI: 1.04-8.28), and operative duration > 360 minutes (adjusted OR: 2.76, 95% CI: 1.01-7.51) were independent predictors of POPF. Patients with POPF had longer hospital stay (18.6 ± 6.2 vs. 11.9 ± 4.1 days) and higher mortality (14.3% vs. 2.8%). However, mortality was likely multifactorial and not solely attributable to POPF.

Conclusion: POPF remains a frequent complication after PD. Soft pancreatic texture, small duct diameter, hypoalbuminemia, elevated CRP, and prolonged operative time are significant independent predictors. Although POPF is associated with adverse postoperative outcomes, its relationship with mortality is likely indirect. Preoperative optimization and meticulous surgical technique may reduce POPF incidence and improve outcomes. Further multicenter studies are required for external validation.

## Introduction

Pancreaticoduodenectomy (PD) remains the standard surgical procedure for benign and malignant diseases involving the pancreatic head and periampullary region. Despite significant advances in surgical techniques, perioperative care, anesthesia, and postoperative management, PD continues to be associated with substantial postoperative morbidity, with complication rates ranging from 30% to 50% [1,2]. Among postoperative complications, postoperative pancreatic fistula (POPF) remains the most clinically significant because of its association with intra-abdominal sepsis, delayed recovery, prolonged hospitalization, increased healthcare costs, and postoperative mortality [3,4].

POPF primarily results from failure of the pancreatico-enteric anastomosis, leading to leakage of pancreatic secretions and subsequent local or systemic complications. To standardize reporting and improve comparability among studies, the International Study Group on Pancreatic Surgery (ISGPS) updated the definition and grading system for POPF in 2016, distinguishing biochemical leak from clinically relevant POPF (grades B and C) according to clinical severity and required interventions [5].

The occurrence of POPF is multifactorial and influenced by patient-related, anatomical, and operative factors. Previous studies have identified soft pancreatic texture, small pancreatic duct diameter, prolonged operative duration, excessive blood loss, perioperative transfusion, and poor nutritional or inflammatory status as major predictors of POPF following PD [6]. Among these, soft pancreatic texture and narrow pancreatic duct diameter are consistently recognized as the strongest operative risk factors because they increase technical difficulty during pancreatico-jejunostomy (PJ) reconstruction [7,8].

Recent studies have also highlighted the importance of systemic inflammatory and nutritional markers in predicting postoperative outcomes after pancreatic surgery [9]. Elevated C-reactive protein (CRP) levels and hypoalbuminemia have been associated with impaired tissue healing, increased infectious complications, and higher rates of clinically relevant POPF [10,11]. In addition, advances in predictive modeling, including nomograms, radiomics, and machine learning approaches, have further emphasized the importance of accurate perioperative risk stratification in patients undergoing PD [12].

Although these risk factors are well established in the global literature, their expression and clinical impact may vary across different healthcare settings. Evidence from low- and middle-income countries, including Bangladesh, remains limited. In our setting, several contextual factors may influence outcomes after PD, including a high prevalence of preoperative malnutrition and hypoalbuminemia, late-stage presentation of disease, selective and variable use of preoperative biliary drainage, differences in perioperative optimization, limited intensive care and monitoring resources, and variability in surgical volume and experience. These differences may alter both the baseline risk profile of patients and the perioperative management strategies compared with high-income settings.

Therefore, this study aimed to evaluate the incidence and risk factors of POPF following PD in a tertiary care center in Bangladesh, with particular emphasis on identifying both established and context-specific determinants relevant to a resource-limited surgical environment.

## Materials and methods

Study design and study population

This prospective observational study was conducted in the Department of Hepatobiliary Surgery, Dhaka Medical College Hospital, Dhaka, Bangladesh, from January 2024 to December 2024. No formal sample size calculation was performed because all eligible patients undergoing PD during the study period were included consecutively. All procedures were performed by a single experienced hepatobiliary surgical team to minimize inter-surgeon variability and ensure technical consistency. Patients were categorized according to the occurrence of POPF based on the 2016 International Study Group of Pancreatic Surgery (ISGPS) criteria [5]. POPF outcomes were reported as biochemical leak, Grade B POPF, and Grade C POPF (Table 1). In addition to the overall POPF analysis, a separate analysis of clinically relevant POPF (CR-POPF), defined as Grade B and Grade C fistulas, was performed.

**Table 1:** International Study Group of Pancreatic Fistula (ISGPF/ISGPS) Definition and Grading of Postoperative Pancreatic Fistula (POPF) POPF: Postoperative pancreatic fistula Source: Ref [5]

Grade	Definition	Clinical Features
Biochemical Leak (formerly Grade A)	Elevated drain amylase level >3 times the upper normal serum amylase value without clinical impact	No change in clinical management; not considered clinically relevant POPF
Grade B	Clinically relevant pancreatic fistula requiring change in postoperative management	Prolonged drainage (>3 weeks), percutaneous/endoscopic intervention, antibiotics, or delayed discharge
Grade C	Severe clinically relevant pancreatic fistula associated with major complications	Organ failure, reoperation, sepsis, or death attributable to POPF

Inclusion criteria

This study included adult patients (aged 18 years and above) who underwent PD for either benign or malignant diseases involving the pancreas or periampullary region.

Exclusion criteria

Patients were excluded if they had unresectable pancreatic or periampullary malignancy identified either preoperatively or during intraoperative assessment. In addition, patients with evidence of distant metastasis were not included. Those deemed unfit for major surgical intervention due to uncontrolled or severe comorbid medical conditions were also excluded from the study.

Data collection

Data were collected prospectively using a structured data collection form. Variables included demographic characteristics, clinical history, laboratory parameters, radiological findings, operative details, and postoperative outcomes.

Preoperative variables included age, sex, body mass index, diabetes mellitus, American Society of Anesthesiologists (ASA) score, serum albumin, serum bilirubin, and preoperative CRP. Information regarding preoperative biliary drainage, neoadjuvant therapy, and final pathology was also recorded.

Pancreatic duct diameter was measured preoperatively using computed tomography (CT) scan and/or magnetic resonance cholangiopancreatography (MRCP) and categorized as < 3 mm or ≥ 3 mm. Pancreatic texture was assessed intraoperatively at the pancreatic transection margin by direct palpation and subjective evaluation of gland consistency by the operating hepatobiliary surgical team and categorized as either "soft" or "hard/fibrotic." Hypoalbuminemia was defined as serum albumin < 32 g/L, and elevated inflammatory status was defined as CRP > 20 mg/L.

Operative variables included operative duration, estimated blood loss (mL), intraoperative blood transfusion requirement, and the type of PJ reconstruction. Estimated intraoperative blood loss (mL) was routinely recorded by the anesthesia and surgical teams using suction volume measurements and assessment of blood-soaked surgical materials. Intraoperative transfusion requirement was analyzed separately as a surrogate marker of operative difficulty and blood loss. Prolonged operative duration was defined as surgery lasting > 360 minutes.

Postoperative outcomes included POPF occurrence and grade, length of hospital stay, in-hospital mortality, 30-day mortality, and 90-day mortality, where available. All postoperative mortality cases were retained in the final statistical analysis and were not excluded at any stage. Patients who died due to POPF-related complications were classified as Grade C POPF and analyzed within the POPF group. Patients who died from unrelated causes without evidence of pancreatic leak were categorized in the non-POPF group. Length of hospital stay was calculated until discharge or death.

Surgical procedure and postoperative management

After preoperative optimization, all patients underwent PD through either a bilateral subcostal or modified Makuuchi incision. Standard resection included cholecystectomy, dissection of the common bile duct, Kocherization of the duodenum, and resection of the pancreatic head and uncinate process.

Pancreatic reconstruction was performed using Blumgart, Cattell-Warren duct-to-mucosa, or dunking PJ techniques. The type of PJ reconstruction was selected intraoperatively according to pancreatic duct diameter, gland texture, remnant pancreatic thickness, and technical feasibility. The Blumgart technique was generally preferred in patients with favorable duct anatomy and satisfactory tissue characteristics, whereas Cattell-Warren duct-to-mucosa or dunking techniques were more commonly used in technically challenging situations such as very small duct diameter, friable pancreatic tissue, or difficult operative exposure.

Hepaticojejunostomy and gastrojejunostomy were subsequently performed. Two closed-suction drains were routinely placed, one near the PJ and one in Morrison's pouch. Drain output volume, character, and amylase concentration were monitored postoperatively. Drain management and postoperative care followed an Enhanced Recovery After Surgery (ERAS) protocol. Oral intake was initiated according to postoperative recovery and advanced as tolerated.

Ethical approval

The study was conducted in accordance with the Declaration of Helsinki and the International Ethical Guidelines for Biomedical Research Involving Human Subjects. Ethical approval was obtained from the Ethical Review Committee of Dhaka Medical College Hospital, Dhaka, Bangladesh (Memo No: DMCH113/2024). Written informed consent was obtained from all participants prior to enrollment. As this was an observational study, trial registration was not required.

Statistical analysis

Data were analyzed using Statistical Product and Service Solutions (SPSS; IBM SPSS Statistics for Windows, Armonk, NY). Continuous variables were expressed as mean ± standard deviation (SD) or median (interquartile range), as appropriate. Categorical variables were expressed as frequencies and percentages.

Comparisons between groups were performed using the independent-samples t-test or Mann-Whitney U test for continuous variables and the chi-square test or Fisher's exact test for categorical variables.

Univariate logistic regression analysis was initially performed to identify variables associated with POPF. Variables demonstrating statistical significance (p < 0.05) and/or strong clinical relevance were considered for inclusion in multivariable logistic regression. To reduce the risk of model overfitting, given the limited number of POPF events, the number of predictors included in the final multivariable model was restricted according to established events-per-variable recommendations. Multicollinearity among candidate predictors was assessed using variance inflation factors (VIFs), and variables demonstrating significant collinearity were excluded from the final model. Results are reported as odds ratios (ORs) with 95% confidence intervals (CIs). A two-sided p-value < 0.05 was considered statistically significant.

## Results

A total of 100 patients who underwent PD during the study period were included in this study. Patients were divided into two groups based on postoperative outcomes: Group A consisted of patients who developed POPF (n = 28), and Group B included patients without POPF (n = 72). The overall incidence of POPF was 28%. According to the 2016 ISGPS classification, biochemical leak was observed in 10 patients (10%), Grade B POPF in 12 patients (12%), and Grade C POPF in six patients (6%). Clinically relevant POPF (Grade B/C) accounted for 18% of the study population.

The mean age of the study population was comparable between the two groups, being 56.9 ± 10.8 years in Group A and 59.4 ± 11.7 years in Group B (p = 0.318). Male predominance was observed in both groups, accounting for 57.1% in the POPF group and 63.9% in the non-POPF group. No statistically significant differences were noted regarding sex distribution, ASA score, primary diagnosis, or associated comorbidities, including diabetes mellitus, ischemic heart disease, chronic kidney disease, and chronic obstructive pulmonary disease/bronchial asthma (Table 2).

**Table 2:** Baseline demographic and clinical characteristics Values are expressed as mean ± standard deviation or number (%). Group A included patients who developed postoperative pancreatic fistula (POPF), while Group B included patients without POPF. Continuous variables were analyzed using Student's t-test, while categorical variables were compared using the chi-square (χ²) test or Fisher's exact test where appropriate. A p-value < 0.05 was considered statistically significant. ASA: American Society of Anesthesiologists; PTBD: Percutaneous transhepatic biliary drainage; COPD: Chronic obstructive pulmonary disease; POPF: Postoperative pancreatic fistula

Variables	Group A POPF (n=28)	Group B Non-POPF (n=72)	Test statistic	p-value
Mean age (years)	56.9 ± 10.8	59.4 ± 11.7	t = -1.02	0.318
Sex			χ² = 0.59	0.441
Male	16 (57.1%)	46 (63.9%)		
Female	12 (42.9%)	26 (36.1%)		
ASA score (mean)	2.4 ± 0.5	2.2 ± 0.6	t = 1.69	0.126
Diagnosis			χ² = 2.24	0.527
Ca head of pancreas	10 (35.7%)	32 (44.4%)		
Periampullary carcinoma	11 (39.3%)	20 (27.8%)		
Distal cholangiocarcinoma	5 (17.9%)	13 (18.1%)		
Others	2 (7.1%)	7 (9.7%)		
Previous intervention (Biliary stenting/PTBD)	16 (57.1%)	30 (41.7%)	χ² = 1.92	0.168
Previous chemoradiotherapy	5 (17.9%)	9 (12.5%)	χ² = 0.47	0.492
Diabetes mellitus	9 (32.1%)	19 (26.4%)	χ² = 0.32	0.574
Ischemic heart disease	4 (14.3%)	8 (11.1%)	Fisher’s exact = 0.18	0.671
Chronic kidney disease	3 (10.7%)	3 (4.2%)	Fisher’s exact = 1.64	0.207
COPD/Bronchial asthma	4 (14.3%)	6 (8.3%)	Fisher’s exact = 0.76	0.383
Other comorbidities	5 (17.9%)	10 (13.9%)	χ² = 0.24	0.623

Preoperative laboratory parameters demonstrated significant differences between the groups. Patients who developed POPF had significantly lower serum albumin levels compared to patients without POPF (30.8 ± 4.2 g/L vs. 34.7 ± 4.8 g/L, p < 0.001). Similarly, mean CRP levels were significantly higher in the POPF group (24.1 ± 10.2 mg/L) compared to the non-POPF group (16.7 ± 7.8 mg/L, p < 0.001). Serum bilirubin levels did not differ significantly between the groups. Radiological assessment revealed that a pancreatic duct diameter of less than 3 mm was significantly associated with POPF formation. Among patients with POPF, 71.4% had a pancreatic duct diameter < 3 mm compared to only 33.3% in the non-POPF group (p < 0.001). Intraoperative findings also demonstrated that soft pancreatic texture was markedly more common among patients who developed POPF (82.1% vs. 43.1%, p < 0.001) (Table 3).

**Table 3:** Comparison of laboratory and radiological parameters Values are presented as mean ± standard deviation or number (%). Continuous variables were analyzed using Student's t-test, while categorical variables were compared using the chi-square (χ²) test. Pancreatic duct diameter was measured preoperatively using computed tomography (CT) scan and magnetic resonance cholangiopancreatography (MRCP). A p-value < 0.05 was considered statistically significant. CRP: C-reactive protein; POPF: Postoperative pancreatic fistula

Variables	Group A POPF (n=28)	Group B Non-POPF (n=72)	Test statistic	p-value
Serum bilirubin (mg/dL)	9.2 ± 5.4	8.1 ± 4.9	t = 0.94	0.347
Serum albumin (g/L)	30.8 ± 4.2	34.7 ± 4.8	t = -4.00	<0.001
C-reactive protein (CRP) (mg/L)	24.1 ± 10.2	16.7 ± 7.8	t = 3.47	<0.001
Diameter of pancreatic duct			χ² = 11.84	<0.001
<3 mm	20 (71.4%)	24 (33.3%)		
>3 mm	8 (28.6%)	48 (66.7%)		

The mean operative duration was significantly longer in patients who developed POPF (392.5 ± 58.3 minutes vs. 341.2 ± 61.7 minutes; p < 0.001). Estimated intraoperative blood loss was also significantly higher in the POPF group (865 ± 295 mL vs. 610 ± 245 mL; p < 0.001). Intraoperative blood transfusion requirement was greater in the POPF group (2.6 ± 1.4 units vs. 1.4 ± 1.0 units; p < 0.001). Soft pancreatic texture was significantly more common among patients with POPF (82.1% vs. 43.1%; p < 0.001). Regarding reconstruction, Blumgart PJ was more frequently performed in the non-POPF group, while Cattell-Warren and dunking techniques were relatively more common in the POPF group (p = 0.041). The selection of reconstruction technique was based on intraoperative pancreatic texture, duct size, anatomic feasibility, and surgeon judgment (Table 4).

**Table 4:** Comparison of intraoperative and postoperative outcomes between patients with and without postoperative pancreatic fistula (POPF) following pancreaticoduodenectomy Values are expressed as mean ± standard deviation or number (percentage). Continuous variables were analyzed using Student's t-test, while categorical variables were compared using the chi-square test or Fisher's exact test. Mortality outcomes include in-hospital, 30-day, and 90-day mortality. A p-value < 0.05 was considered statistically significant. PJ: Pancreatico-jejunostomy

Variables	Group A POPF (n=28)	Group B Non-POPF (n=72)	Test statistic	p-value
Operative variables				
Duration of operation (min)	392.5 ± 58.3	341.2 ± 61.7	t = 3.89	<0.001
Estimated blood loss (mL)	865 ± 295	610 ± 245	t = 4.21	<0.001
Intraoperative transfusion (units)	2.6 ± 1.4	1.4 ± 1.0	t = 4.14	<0.001
Soft pancreatic texture	23 (82.1%)	31 (43.1%)	χ² = 12.30	<0.001
Pancreatico-jejunostomy technique			χ² = 6.36	0.041
Blumgart	10 (35.7%)	42 (58.3%)		
Cattell-Warren	11 (39.3%)	19 (26.4%)		
Dunking	7 (25.0%)	11 (15.3%)		
Postoperative outcomes				
Length of hospital stay (days)	18.6 ± 6.2	11.9 ± 4.1	t = 5.29	<0.001
Mortality outcomes				
In-hospital mortality	4 (14.3%)	2 (2.8%)	Fisher = 4.83	0.028
30-day mortality	5 (17.9%)	3 (4.2%)	Fisher = 5.11	0.024
90-day mortality	6 (21.4%)	4 (5.6%)	Fisher = 5.73	0.017

Patients with POPF had significantly worse postoperative outcomes. The mean hospital stay was longer in Group A compared to Group B (18.6 ± 6.2 days vs. 11.9 ± 4.1 days; p < 0.001). Overall, in-hospital mortality was 6% (6/100). Mortality was significantly higher in the POPF group (14.3% vs. 2.8%). The 30-day mortality was 8% (8/100), and the 90-day mortality was 10% (10/100) (Table 4). Deaths in the POPF group were mainly due to intra-abdominal sepsis with multiorgan failure (n = 5) and post-pancreatectomy hemorrhage (n = 1). In the non-POPF group, deaths were due to myocardial infarction (n = 2) and respiratory failure secondary to pulmonary complications (n = 2). In Hospital mortality cases were classified according to ISGPS criteria; deaths directly related to pancreatic fistula were categorized as Grade C POPF.

Univariate logistic regression analysis identified several significant risk factors associated with POPF development. Soft pancreatic texture demonstrated the strongest association with POPF (OR: 6.04, 95% CI: 2.16-16.87, p < 0.001). Pancreatic duct diameter < 3 mm was also significantly associated with increased risk of POPF (OR: 5.00, 95% CI: 1.97-12.66, p < 0.001). Other significant factors included elevated CRP > 20 mg/L (OR: 4.27, 95% CI: 1.71-10.64, p = 0.002), serum albumin < 32 g/L (OR: 3.84, 95% CI: 1.52-9.68, p = 0.004), prolonged operative duration > 360 minutes (OR: 3.41, 95% CI: 1.39-8.34, p = 0.007), and intraoperative transfusion > 2 units (OR: 2.89, 95% CI: 1.13-7.39, p = 0.026) (Table 5).

**Table 5:** Univariate logistic regression analysis of risk factors for POPF Results are expressed as odds ratios (OR) with corresponding 95% confidence intervals (CI). Variables included preoperative laboratory parameters, radiological findings, and intraoperative factors. A p-value < 0.05 was considered statistically significant. Estimated blood loss was significantly higher in the POPF group; however, it was excluded from the final logistic regression model due to collinearity with intraoperative transfusion requirement. CRP: C-reactive protein; PJ: Pancreatico-jejunostomy; POPF: Postoperative pancreatic fistula

Variables	Odds Ratio (OR)	95% CI	p-value
Serum albumin <32 g/L	3.84	1.52-9.68	0.004
CRP >20 mg/L	4.27	1.71-10.64	0.002
Pancreatic duct <3 mm	5	1.97-12.66	<0.001
Soft pancreatic texture	6.04	2.16-16.87	<0.001
Operation time >360 min	3.41	1.39-8.34	0.007
Intraoperative transfusion >2 units	2.89	1.13-7.39	0.026
Dunking PJ technique	2.67	1.01-7.02	0.047

On multivariate logistic regression analysis, soft pancreatic texture (adjusted OR: 5.21, 95% CI: 1.73-15.68, p = 0.003), pancreatic duct diameter < 3 mm (adjusted OR: 4.36, 95% CI: 1.52-12.47, p = 0.006), hypoalbuminemia (adjusted OR: 3.02, 95% CI: 1.11-8.19, p = 0.031), elevated CRP (adjusted OR: 2.94, 95% CI: 1.04-8.28, p = 0.041), and prolonged operative dration (adjusted OR: 2.76, 95% CI: 1.01-7.51, p = 0.048) remained independent predictors of POPF following PD (Table 6).

**Table 6:** Multivariate logistic regression analysis identifying independent predictors of postoperative pancreatic fistula (POPF) following pancreaticoduodenectomy Multivariate logistic regression analysis was performed using variables that showed statistical significance in univariate analysis while minimizing multicollinearity. A p-value < 0.05 was considered statistically significant. Adjusted OR: Adjusted odds ratio; CI: Confidence interval; CRP: C-reactive protein

Variables	Adjusted OR	95% Confidence Interval (CI)	p-value
Pancreatic duct diameter <3 mm	4.36	1.52-12.47	0.006
Soft pancreatic texture	5.21	1.73-15.68	0.003
Serum albumin <32 g/L	3.02	1.11-8.19	0.031
CRP > 20 mg/L	2.94	1.04-8.28	0.041
Duration of surgery >360 min	2.76	1.01-7.51	0.048

## Discussion

POPF remains one of the most clinically significant complications following PD and continues to contribute substantially to postoperative morbidity, prolonged hospitalization, and increased healthcare costs. In the present study, POPF occurred in 28% of patients, with clinically relevant POPF (Grade B/C) accounting for the majority of clinically significant outcomes. This incidence is consistent with previously reported rates in contemporary literature, although variations in reporting standards, POPF definitions, and patient selection may account for differences across studies [13,14].

Importantly, while multiple studies have identified risk factors for POPF, the present study does not aim to establish novel predictors, but rather to evaluate the applicability of established risk factors within a Bangladeshi tertiary-care setting. In low- and middle-income countries, such as Bangladesh, patient profiles often differ significantly from those in high-income settings, particularly in terms of nutritional status, late disease presentation, biliary obstruction, preoperative biliary drainage practices, and perioperative resource availability. These contextual differences may influence both baseline risk and postoperative outcomes following PD, thereby providing region-specific clinical relevance to the present findings.

Among all evaluated variables, soft pancreatic texture emerged as the strongest independent predictor of POPF. Patients with soft pancreatic parenchyma had significantly higher odds of developing POPF. However, it is important to interpret this finding as an association rather than a causal relationship, as pancreatic texture may also reflect underlying disease pathology, glandular exocrine activity, and intraoperative technical complexity. This finding is consistent with previous studies and meta-analyses demonstrating that soft pancreas is a major determinant of clinically relevant POPF due to increased fragility and difficulty in achieving secure anastomosis [15,16].

Similarly, a pancreatic duct diameter of < 3 mm was independently associated with POPF. Smaller duct size increases the technical complexity of duct-to-mucosa anastomosis and may reduce the accuracy of suture placement, thereby increasing the risk of pancreatic leakage [17]. Omar et al., in a prospective cohort study, similarly identified small pancreatic duct diameter as an independent operative risk factor for clinically relevant POPF following PD [18]. These findings further support the importance of careful intraoperative assessment of duct size during pancreatic reconstruction. However, duct size is also closely associated with underlying disease biology and pancreatic fibrosis, which may confound its direct effect. Therefore, duct diameter should be interpreted as a marker of operative complexity rather than an isolated causal factor.

Preoperative hypoalbuminemia was also independently associated with POPF. Serum albumin reflects both nutritional status and systemic inflammatory burden. In the present cohort, hypoalbuminemia likely reflects the high prevalence of malnutrition and late-stage disease presentation in our population. These findings reinforce the importance of preoperative nutritional assessment and optimization, particularly in resource-limited settings. Malnutrition may impair collagen synthesis, tissue regeneration, and immune function, thereby negatively affecting anastomotic healing. Previous studies evaluating pancreatic surgery outcomes have similarly demonstrated increased postoperative complications among patients with low serum albumin levels [19,20]. However, hypoalbuminemia should not be interpreted in isolation, as it may also represent underlying malignancy-related cachexia or obstructive jaundice.

Elevated preoperative CRP was significantly associated with POPF. It is essential to distinguish that CRP in this study was measured preoperatively, unlike several studies that evaluate postoperative CRP as an early predictive biomarker [21,22]. Elevated preoperative CRP may reflect underlying cholangitis, biliary stenting, tumor-related inflammation, or systemic inflammatory response. These conditions may adversely affect tissue healing and predispose to postoperative complications. Therefore, CRP should be interpreted as a marker of systemic inflammatory state rather than a direct causal determinant of POPF.

Prolonged operative duration (> 360 minutes) was also independently associated with POPF. This likely reflects increased surgical complexity, difficult dissection, intraoperative technical challenges, and tissue handling. Previous studies evaluating PD outcomes have similarly shown that prolonged operative time is associated with increased postoperative morbidity and infectious complications [23,24]. Efficient operative technique and minimization of unnecessary intraoperative delays may therefore contribute to reducing POPF incidence. However, operative time should be considered a surrogate marker rather than an independent biological cause of fistula formation.

Although intraoperative blood transfusion requirement was significantly associated with POPF on univariate analysis, it did not remain independently significant after multivariate adjustment. Increased transfusion requirement often reflects technically difficult surgery or excessive blood loss and has previously been associated with postoperative immunosuppression and infection [25]. Meticulous surgical dissection and careful hemostasis remain essential to minimizing intraoperative blood loss and improving postoperative outcomes.

The role of the PJ technique remains controversial. In this study, the Blumgart technique appeared to be associated with lower POPF rates in unadjusted analysis; however, it did not remain an independent predictor after adjustment. Previous comparative studies evaluating different PJ techniques have reported inconsistent findings, with no universally superior reconstruction method identified [26]. Importantly, the choice of reconstruction technique in our institution was not randomized but based on intraoperative findings, including pancreatic texture, duct size, and surgeon judgment. Therefore, the observed differences likely reflect selection bias rather than the true superiority of one technique over another. This limitation is important when interpreting surgical technique-related outcomes.

The clinical impact of POPF in this study was substantial. Patients who developed POPF had significantly higher morbidity, prolonged hospital stay, and increased mortality. However, it is critical to emphasize that POPF is likely a marker of overall surgical complexity and patient vulnerability rather than the sole direct cause of mortality in all cases. In several instances, deaths were related to sepsis, hemorrhage, and multiorgan failure, conditions that may be triggered or exacerbated by POPF but also influenced by baseline patient status and operative difficulty. Therefore, the relationship between POPF and mortality should be interpreted as an association rather than a direct causal pathway.

The main strength of this study lies in providing prospective data from a Bangladeshi tertiary-care center, where limited published evidence exists regarding pancreatic surgery outcomes. Differences in nutritional status, healthcare resources, perioperative optimization, and surgical volume may influence outcomes compared to high-income settings. Thus, while the risk factors identified are consistent with global literature, their confirmation in a resource-limited environment adds important regional value.

This study has several limitations. First, the single-center design and relatively small sample size may limit generalizability. Second, the subjective assessment of pancreatic texture introduces potential interobserver variability. Third, the non-randomized selection of the PJ technique introduces selection bias. Fourth, the limited number of POPF events raises the possibility of model overfitting in multivariate analysis. Fifth, biochemical leak and clinically relevant POPF (Grade B/C) were analyzed separately, but subgroup-specific mechanistic differences could not be fully explored due to sample size constraints. Finally, mortality attribution remains limited by retrospective classification of clinical causes.

## Conclusions

POPF is a frequent complication following PD, observed in 28% of patients in this study. Soft pancreatic texture, small duct size (< 3 mm), hypoalbuminemia, elevated CRP, and prolonged operative time were independent predictors of POPF and may assist in early risk stratification. Although POPF was associated with higher postoperative morbidity and mortality, causal inference cannot be established from this observational study. The observed mortality is likely multifactorial and related to underlying patient condition and operative complexity rather than POPF alone. Focused preoperative optimization, careful surgical technique, and vigilant perioperative care may help reduce POPF incidence. Larger prospective studies are required to further clarify these associations and improve predictive accuracy.
